# Research Trends in the Development of Block Copolymer-Based Biosensing Platforms

**DOI:** 10.3390/bios14110542

**Published:** 2024-11-08

**Authors:** Yong-Ho Chung, Jung Kwon Oh

**Affiliations:** 1 Department of Chemical Engineering, Hoseo University, Asan-si 31499, Republic of Korea; 2 Department of Chemistry and Biochemistry, Concordia University, Montreal, QC H4B 1R6, Canada

**Keywords:** biosensors, block copolymers, self-assembly, biosensing platforms, nanostructures

## Abstract

Biosensing technology, which aims to measure and control the signals of biological substances, has recently been developed rapidly due to increasing concerns about health and the environment. Top–down technologies have been used mainly with a focus on reducing the size of biomaterials to the nano-level. However, bottom–up technologies such as self-assembly can provide more opportunities to molecular-level arrangements such as directionality and the shape of biomaterials. In particular, block copolymers (BCPs) and their self-assembly have been significantly explored as an effective means of bottom–up technologies to achieve recent advances in molecular-level fine control and imaging technology. BCPs have been widely used in various biosensing research fields because they can artificially control highly complex nano-scale structures in a directionally controlled manner, and future application research based on interactions with biomolecules according to the development and synthesis of new BCP structures is greatly anticipated. Here, we comprehensively discuss the basic principles of BCPs technology, the current status of their applications in biosensing technology, and their limitations and future prospects. Rather than discussing a specific field in depth, this study comprehensively covers the overall content of BCPs as a biosensing platform, and through this, we hope to increase researchers’ understanding of adjacent research fields and provide research inspiration, thereby bringing about great advances in the relevant research fields.

## 1. Introduction

Block copolymers (BCPs) are large polymer structures in which monomers with different chemical structures are covalently connected to each other according to a certain rule [1,2]. Each monomer has different physical and chemical properties. When these monomers are specifically structured, very unique and characteristic copolymers can be synthesized [3,4,5,6,7]. Depending on their architectures, they can be classified into various polymeric structures such as linear, branched, circular, core–shell, star, and spike structures. The synthesis techniques are very diverse, but they can be broadly divided into polymerization techniques that sequentially add monomers to the main chain and methods that utilize the interaction between specific functional groups located at the end of the polymer chain [8,9,10,11,12].

Thanks to the development of such synthetic technologies, since the 2000s, a variety of BCPs have been synthesized and utilized for the construction of functional new materials. The most basic application is thermoplastic elastomers, and BCPS technology has been utilized in molding, extrusion, etc. related to the development and commercialization of various functional plastics [13,14]. In addition, they have been explored as nanocarriers for drug delivery into the body, soft lithography, and porous materials [15,16,17].

Further, BCPs have been actively applied and utilized in the development of various biosensors over the past several decades, due to their functional and structural diversity [2,18,19]. The field of biosensors has long attracted the attention of research and industry [20,21,22]. Recently, the research trend has gradually shifted toward miniaturization, high performance, and wearable forms [23,24,25]. Considering the most basic operating principles of biosensors and recent research trends, the most important point to consider is the physical measurement platform that is required to transform biological substances such as biomarkers into measurable forms, such as electrical and optical signals.

To provide such physical platforms, solid electrode formation technologies such as photolithography, e-beam lithography, and nanoimprinting have been explored [26,27,28]. Moreover, various metal-based nanoparticles such as gold/silver and quantum dots have been used to create platforms for the immobilization of biomaterials, signal amplification, and control [29,30,31]. However, in order to satisfy recent miniaturization, ultra-high sensitivity, and biocompatibility, a bottom–up approach rather than a top–down approach is required. The top–down approach refers to the method of gradually reducing the size of nanostructures, and a representative example is the semiconductor process. On the other hand, the bottom–up approach is to control molecular-level building blocks to produce larger structures, and a representative example is the self-assembly technique of molecules. BCPs that can directionally control the binding of monomers can provide a new alternative. BCPs can be used alone for nanopatterning, or they can be used for sensing applications that utilize structural changes based on reactivity according to the environment by forming big particles such as vesicles and micelles at the nano- and micro-level. In addition, combined with the existing top–down technology, signal sensitivity and precision can be further improved.

As a result, BCPs could provide an original methodological technology for artificially synthesizing very delicate nanostructures at the molecular level. This technology can not only be a complementary substitute for the solid substrate-based nanopatterning technology utilizing the existing semiconductor process but also has greater significance as a platform technology for detecting, controlling, and regulating biomaterials of several nanometers. In particular, in the operation and characteristic expression of biomolecules, the control of the size below nanometers, as well as the control of direction and location, are very important factors. At this time, the unique and versatile ability of BCPs to generate artificial structural complexity can efficiently support this, which has greatly promoted the development of the biosensor field to date, and in the future, new synthetic strategies and implementations based on BCPs and various application studies according to combination with biomolecules are greatly anticipated.

BCPs have been studied extensively for a long time, and the results of fundamental research on the principles and processes of synthesizing artificial nanostructures using BCPs, interactions between molecules at the nano-scale including biomolecules, and various application studies have been published. In addition, various review papers that provide insight into these research results have been published, and they mainly consist of in-depth discussions on specific topics such as the synthesis of BCPs, the interfaces between BCPs and molecules, and their applications in specific fields. This review is unique in that it comprehensively covers the entire biosensing platform based on BCPs. It focuses on breadth rather than depth in the research field, and through this, we believe that various researchers will be able to gain research insights by acquiring information on adjacent related fields through this review paper.

In this review, we provide a general overview of recent research on biosensing platforms based on BCPs and discuss the future direction of research. This review is outlined by the structural formation based on self-assembly, which is the basic principle of BCPs, in Section 2, the biosensing platform applications of BCPs by type in Section 3, other applications of BCPs in Section 4, and finally a conclusion and future prospects in Section 5.

## 2. Self-Assembly-Based Nanostructure Formation of BCPs

BCPs have a polymer structure with repeating identical complex structures, and by utilizing these structural features, nano-level patterns with controllable directionality and shape can be produced. Nanopatterns produced in this way are very economical and efficient compared to top–down techniques based on semiconductor processes because they utilize self-assembly characteristics. These patterns can be utilized as direct reaction surfaces depending on the electrical and chemical characteristics of the monomers used in the synthesis of BCPs and can also serve as precursors for producing conductive material patterns such as metal materials.

### 2.1. Initial Synthesis of BCPs

BCPs are basically manufactured by combining two or more monomers with different chemical properties. The initial synthesis is mainly performed in a bulk solution, and the commonly used synthesis techniques are usually controlled radical polymerization (CRP) and living anionic polymerization. The former is the most common synthesis technique in which unsaturated monomer molecules are sequentially added to the free radical reactive sites at the terminal, and it can be specifically divided into atom transfer radical polymerization (ATRP), reversible addition/fragmentation chain transfer polymerization (RAFT), and nitroxide-mediated polymerization (NMP). The latter is a reaction in which polymerization continues without a termination reaction, and it is used to obtain BCPs with the most well-defined structure because it makes it easy to control architecture, composition, and molecular weight compared to the CRP method. In addition, there are methods that combine various polymer techniques.

In addition to cross-linking reactions such as the sequential addition polymerization method described above, micromolecular reactions such as polymer-analogous transformations or interchain exchange reactions are important and promising technologies used to synthesize desired copolymers [32,33]. The former is a method to modify substituents without affecting the molecular weight distribution or polymerization degree of the polymer structure itself [34,35], and the latter is a method to synthesize new polymers through chain exchange between uniform polymers; for example, two types of condensation polymers A and B can be synthesized into the block copolymer AB through the corresponding reaction [36,37].

**Figure 1:**
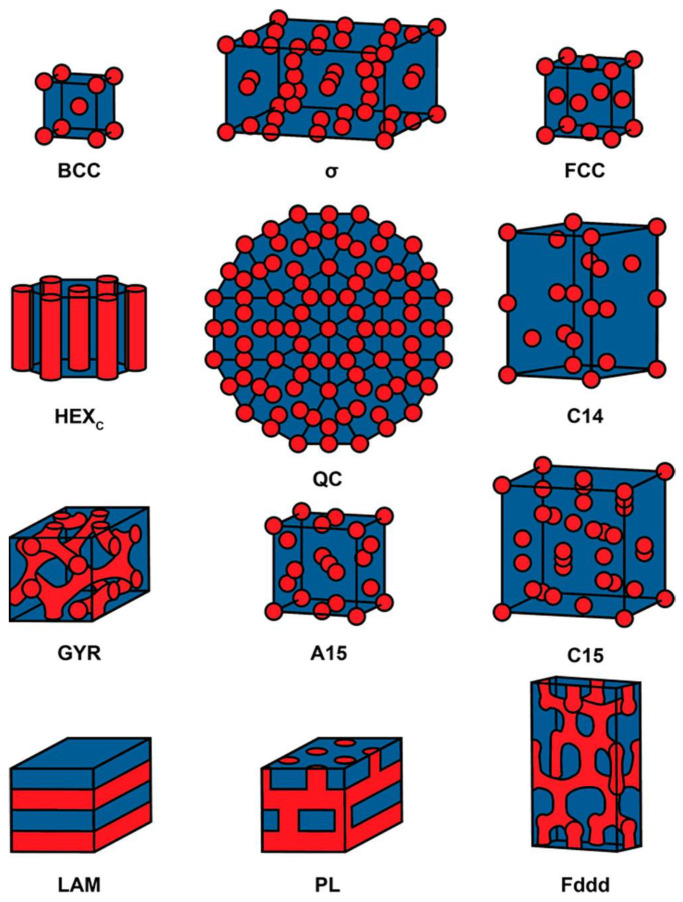
Nanostructures reported for linear AB diblock copolymers. BCC: body-centered cubic; σ: Frank–Kasper sigma phase; FCC: face-centered cubic; HEXc: hexagonally packed cylinders; QC: dodecagonal quasi-crystal; C14: Frank–Kasper AB2 Laves phase; GYR: double gyroid; A15: Frank–Kasper AB3 phase; C15: Frank–Kasper AB2 Laves phase; LAM: lamellae; PL: perforated lamellae; Fddd: O70 network. Reprinted with permission from [46], Copyright [2020], American Chemical Society.

And, in the synthesized BCPs, two or more chemically different polymer chains are covalently linked and are divided into diblocks, triblocks, and multiblocks depending on the number of chains. The individual chains that make up most BCPs exhibit incompatibility, and the compatibility increases as the temperature increases. Therefore, in the low temperature region, they exist separated in microphases due to the repulsion between chains that cannot be mixed with each other, and as the temperature increases, this repulsive force decreases and they are converted into a homogeneous phase. The temperature at which the microphase is converted into a homogeneous phase is called the ordered-to-disordered transition temperature (T_ODT_). This phenomenon is determined by the balance of forces related to the separation and mixing of functional groups, and the variables related to this include the Flory–Huggins interaction parameter (χ), the degree of polymerization (N), and the ratio of polymer unit blocks (f), and many theoretical and experimental studies have been conducted on this [2,38,39,40,41]. As a result, BCPs typically exhibit morphologies such as spheres, cylinders, gyroids, and lamellae [42,43,44,45] and can also be configured in various detailed shapes, as shown in the example in Figure 1 [46].

### 2.2. Nanopatterning Utilizing the Self-Assembly Properties of BCPs

Self-assembly technology, one of the representative bottom–up synthesis methods, can create nanostructures by gradually stacking them by controlling the arrangement or bonding between molecules. This method can provide great advantages, especially in the field of nanopatterning, compared to the top–down method in terms of economy due to the low difficulty of the process itself and efficiency; it can be carried out on a large scale in bulk. The overall bonding form and arrangement of BCPs can be controlled depending on the composition of the internal monomers, the type of solvent, and the process conditions. In addition, the variables that determine the morphologies and sizes of BCP-based nanopatterns include the volume fraction of the constituent polymers, the degree of polymerization (N), and the level of incompatibility between blocks (Flory–Huggins interaction parameter, χ) [40,47]. In particular, polymers with very high χ are capable of forming microphase-separated domains with very small feature sizes of less than 10 nm, and thus are of great interest in the field of nanopatterning [48].

Lithography using these characteristics theoretically enables patterning of less than 10 nm, and experimental results of less than that have been reported [49,50,51]. However, stable patterning that can be practically used still remains a challenge [52].

**Figure 2:**
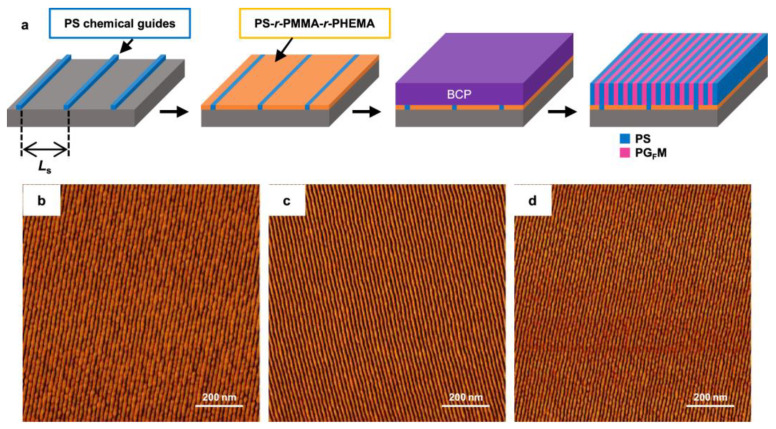
Fabricating sub-20 nm line patterns by DSA using PS-b-PGFM. (**a**) Schematic of the DSA process using a PS-b-PGFM on a chemically patterned Si substrate; AFM phase images of (**a**,**b**) PS-b-PGFM19-23 film on an NL35-modified DSA substrate (Ls = 90 nm) after annealing at 240 °C for 5 min; (**c**) PS-b-PGFM19-10 film on an NL38-modified DSA substrate (Ls = 84 nm) after annealing at 230 °C for 5 min; and (**d**) PS-b-PGFM18-11 film on an NL38-modified DSA substrate (Ls = 90 nm) after annealing at 230 °C for 5 min. All the thin films are 19 nm thick and were etched using O2 plasma for 10 s prior to AFM. Reprinted with permission from [47], Copyright [2024] by the authors (CC BY 4.0).

Recently, Maekawa et al. reported the results of a study that could stably form sub-10 nm patterns by modifying the structure of polystyrene-block-poly(methyl methacrylate) (PS-b-PMMA) [47]. PS-b-PMMA has attracted much attention, not only as a target material for next-generation lithography through combination with extreme ultraviolet lithography processes, but also in other directed self-assembly (DSA)-based applications [53,54]. As shown in Figure 2, the PMMA portion can be modified to have various ratios of poly(glycidyl methacrylate) (PGMA) using the thiol–epoxy reaction, and synthesized BCPs with a Flory–Huggins interaction parameter (χ) that is about four times higher than that of conventional PS-b-PMMA can successfully synthesize sub-10 nm-level patterns stably and reliably through this.

PS-d-PMMA is the most studied and interesting material because it can be easily controlled by heat to produce layered or cylindrical structures. The advantages of this material include that both PS and PMMA have almost the same surface energy at annealing temperatures (190–230 °C), the PMMA domains can be selectively removed, and random copolymers can be used to control interfacial interactions [52,55,56]. For these reasons, various studies have been reported on achieving nano-scale patterns using BCPs based on different monomers [57,58,59].

Shastry et al. reported a method for fabricating various nanostructured thin films and multilayered nanopatterns using the solvent annealing of polystyrene-block-polydimethylsiloxane (PS-b-PDMS) [60]. A multilayer structure was fabricated by controlling and stacking the same type of BCPs to have different arrangements using solvent annealing, and these results can provide clues for the fabrication of controllable three-dimensional nanopatterns. F Feng et al. reported a nanopatterning technology on various solid surfaces by utilizing the self-brushing function, which is a directional self-assembly through the structural functionalization of polystyrene-block-poly(glycidyl methacrylate) (PS-b-PGMA) [61].

## 3. Biosensing Applications Based on BCPs

In biosensing applications, there are various approaches that directly or indirectly utilize the structural features of BCPs. They can be used in the form of physical platforms such as membranes or nanochannels to directly distinguish and detect or control biomaterials. Also, as the surrounding operating environment changes, the combined structure of the monomer changes, causing physical and chemical changes, allowing biosensing functions to be performed. In addition, an indirect approach is also possible to control the arrangement of the combined biomaterials to increase the reactivity or reliability by utilizing the characteristics that can structurally control the direction or position of the functional group. BCPs are mainly used directly or indirectly based on self-assembly technology to produce nanostructures for biosensing platforms. In the case of conventional methods, inorganic solid-based electrodes and platforms as well as metal particles are mainly used. Although the advantages of BCPs compared to conventional materials cannot be discussed in terms of arithmetical and absolute criteria, conventional methods usually use toxic processes to process and discard inorganic-based materials, the materials are relatively expensive, and the manufacturing process is complex. On the other hand, the BCPs-based process is a solution-based bulk process that is relatively safe, uses inexpensive materials, and utilizes self-assembly, so the process is simple, and nanostructures with various structures and morphologies can be produced. It also has the advantage of being able to be performed in large quantities. Table 1 briefly summarizes the types of applications and their corresponding features, the roles of BCPs, and references.

### 3.1. Traditional Signal Transduction Biosensors

The concept of a traditional or conservative biosensor refers to a device that can detect signal changes before and after a target substance that requires analysis on a sensing platform by using a biological reaction pathway. The target substances are diverse, such as proteins, DNA, cells, or organic and inorganic substances that require monitoring, and the sensing platforms are configured in various forms according to the type of conversion signal, such as electrochemical, optical signals, and temperature, to detect them [117,118,119].

Electrochemical biosensors, one of the oldest biosensing technologies, can diagnose biological substances using redox reactions or electrical conductance changes and have been widely used in clinical diagnosis until recently [120,121,122,123]. One of the most important factors for measuring biomaterial signals reliably and sensitively through an electrochemical method is the functionality of the solid electrode, which acts as an interface for electron transfer between the measuring device and the biological substance and its compatibility with the biological substance. For this purpose, carbon-based materials such as carbon nanotubes or graphene [62,124], metal-based materials [63], and other materials have been studied in various ways as target electrode candidates. Although the basic electron transfer properties of the material itself are important, the first thing to consider is the absolute size of the electrode surface area for signal detection. Therefore, irregular structures or porous structures with uneven surfaces have been introduced to increase the electrode area available for the reaction and thus improve the efficiency of electron transfer. For example, Liu et al. applied three-dimensionally manufactured copper nanowires and copper oxide nanoflowers to a glucose sensor to increase the electrode area required for the reaction and thus improved the electron transfer efficiency, achieving a high sensitivity of 32,330 μA mM^−1^ cm^−2^ and a very low detection limit of 20 nM [64].

**Figure 3:**
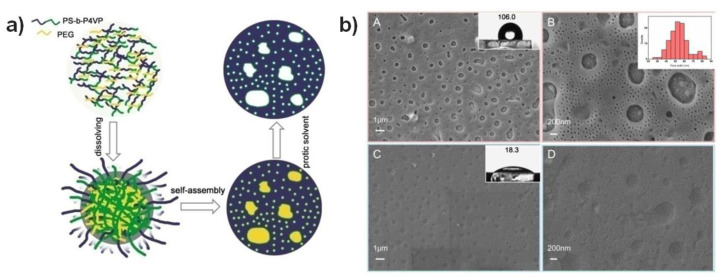
(**a**) Schematic illustration for the fabrication of the hierarchically porous PS-*b*-P4VP film; (**b**) SEM images of PS-*b*-P4VP film (**A**,**B**) and GOx/PS-*b*-P4VP composite film (**C**,**D**) with different magnifications; contact angle images of PS-*b*-P4VP film (inset A) and GOx/PS-*b*-P4VP composite film (inset C); the size distribution of nano-pores in PS-*b*-P4VP film (inset B). Reprinted with permission from [68], Copyright [2018] by the authors (CC BY 4.0).

BCPs have self-assembly properties, and by utilizing this, various micro- and nano-porous materials with selective orientation and structure can be fabricated for use as electrochemical biosensors [65,66,67]. Guo et al. fabricated a glucose sensor using a hierarchical porous structure based on polystyrene-block-poly(4-vinyl pyridine) (PS-b-P4VP) and poly-ethylene glycol (PEG) and reported a long linear range of 10–4500 μM and a low detection limit of 0.05 μM. As shown in Figure 3, the hole size of the fabricated porous structure was in the tens of nanometers, which is advantageous for enzyme immobilization of about 10–100 nm, and it can provide a better operating environment for the immobilized enzyme by securing a hydrophilic surface as well as increasing the reaction surface area [68]. Similarly, Jia et al. developed a highly reliable and stable hydrogen peroxide detection sensor by immobilizing hemoglobin using poly(ethylene oxide)–poly(propylene oxide)–poly(ethylene oxide) (PEO–PPO–PEO) triblock copolymers to maintain the bioactivity of the protein while improving the electron transfer efficiency [125]. In addition to enzyme immobilization, the results of a study using BCPs-based nanostructures for the amplification of the signal generated from enzymes were also reported [126]. In that study, the signal generated from the enzyme was amplified by linking gold nanoparticles with BCPs based on polypyrrole and Pluronic F127. Meanwhile, the results of an electrochemical DNA detection study using a nano-porous film made of BPCs were also published [127]. Folded probe DNA was first immobilized on the electrode surface, and the principle of unfolding when complementary DNA is specifically bound was utilized. By controlling the dynamic properties of the DNA and enhancing hybridization, the sensitivity of the DNA detection was much higher within the porous structure of BPCs.

Optical biosensors have the characteristic of using an optical converter in the signal measurement step and can perform highly selective, sensitive, and rapid measurements compared to other biosensing platforms [69,128]. In addition, they can be classified into label-based and label-free methods depending on the direct use of a label in detecting the optical signal [129,130]. BCPs are also widely applied to optical biosensors. For example, a study on the detection of BSA protein using silica optical fiber functionalized with an amphiphilic block copolymer was reported [70]. The structure of the optical fiber surface changes due to external factors such as protein adsorption, which changes the refractive index of the optical fiber and the thickness of the film, causing fluctuations in the output power measured at the end of the optical fiber and thus enabling the sensitive detection of target substances. In addition, fluorescent biosensors utilizing nanoparticles have been widely studied recently [71,72], and BCPs can provide a great advantage in synthesizing nanoparticles because they allow for precise structural control [73,74]. Liu et al. fabricated fluorescent block copolymer nanoparticles by mixing block copolymer and fluorescent conjugate polymer and showed an excellent selectivity and very high sensitivity of 1.5 ng/mL for tyrosinase (TRY) activity detection [75]. Similarly to fluorescent biosensors, many research results have been published on colorimetric biosensors that recognize the state change in a target substance through color changes. As a colorimetric sensor application for general environmental change detection, Lee et al. reported a sensor that recognizes temperature change through four color changes in the temperature range of 25 to 45 °C using three types of fluorescent blocks based on graphene oxide–BCPs composites [131], and a colorimetric sensor that measures pH change using a similar operating principle was also developed by the same research group [76]. As an application for bioenvironment monitoring, the development of a temperature-sensitive BCPs-based microgel for monitoring temperature changes due to photothermal heating in biological fluids was also reported, which can also detect temperature changes through color changes [77]. In addition, optical biosensors with more sensitive biosignal change detection technologies have been extensively studied. Representative examples include localized surface plasmon resonance (LSPR) [78,79,132], which utilizes the resonance phenomenon of metal nanoparticles, and surface-enhanced Raman scattering (SERS) [133,134,135], which uses Raman signal amplification with a similar principle, and much research is still in progress.

In addition, various types of signal transduction biosensors, such as piezoelectric and calorimetric methods, have been reported. The former measures the change in resonant frequency according to the mass change when a substance is attached to a piezoelectric crystal by affinity interaction [80,81,82], and various studies have reported using molecularly imprinted polymers (MIPs) technology based on polymers for affinity control with the attached substance [83,136,137]. The latter are thermal biosensors that detect temperature changes and directly detect the heat of a 20–100 KJ/mol substrate released from a typical enzymatic reaction [84,85]. The reaction heat of such substrate–enzyme reactions is mainly measured by thermoelectric sensors and is widely used in biosensors for diagnosing various target substances based on this [138,139,140]. In addition, various studies have been conducted to improve the activity and stability of the reaction by introducing BCPs to the interface region between the enzyme, which is the main reactant, and the solid electrode for sensing [141,142,143], and it is expected that various application studies will be conducted in the future in conjunction with temperature-responsive BCPs [144,145].

### 3.2. NanoChannels for Physical Movement and Separation

Channel proteins, which are responsible for the transport of substances at the ion or molecular level in the body, perform a wide variety of functions through nano-level passage control [146,147], and various application studies are being conducted to mimic these functions, such as ion-selective channels, selective drug delivery, and the molecular-level separation of proteins [86,148,149]. Various methods for fabricating nanochannels for these applications have been reported, but most of the solid electrode-based methods have very complicated processes and have difficulties in fabrication due to the use of hazardous materials [87,88]. As an alternative, a method for fabricating nanochannels using BCPs has been reported, and it is receiving much attention due to its ability to easily control the channel size and the relatively simple fabrication process.

**Figure 4:**
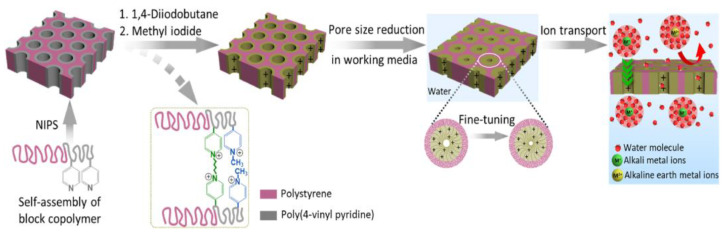
Schematic illustration of isoporous membrane fabrication with polystyrene-block-poly(4-vinylpyridine) (PS-b-P4VP), the post-modifications with alkyl halides 1,4-diiodobutane (DIB) and methyl iodide (MeI), the fine-tuning of pore size, and the ion transport behavior. Reprinted with permission from [89], Copyright [2024] by the author (CC BY 4.0).

Zhang et al. fabricated positively charged isoporous soft nanochannels using PS-b-P4VP [89]. As shown in Figure 4, the fabricated nanochannels were several tens of nanometers in size, and the pore size could be controlled by adjusting the swelling of the pore-forming block in the working media. Using the fabricated nanochannels, ionic substances such as Mg^2+^ could be selectively and successfully separated. These nanochannels can be utilized in nanofiltration membranes that can separate various substances with a size of several nanometers and have been widely applied to the separation and identification of substances such as fine proteins [90,91,92]. For example, a study on protein separation using PS-b-P4VP nanochannels that can be manufactured by adjusting the size from 17 nm to 53 nm was reported [150], and a study on separating hemoglobin (6.4 nm × 5.5 nm × 5 nm) and BSA (7 nm × 3.8 nm × 3.8 nm), which have similar sizes, using nanochannels manufactured with a sub-10 nm size was also reported [151].

**Figure 5:**
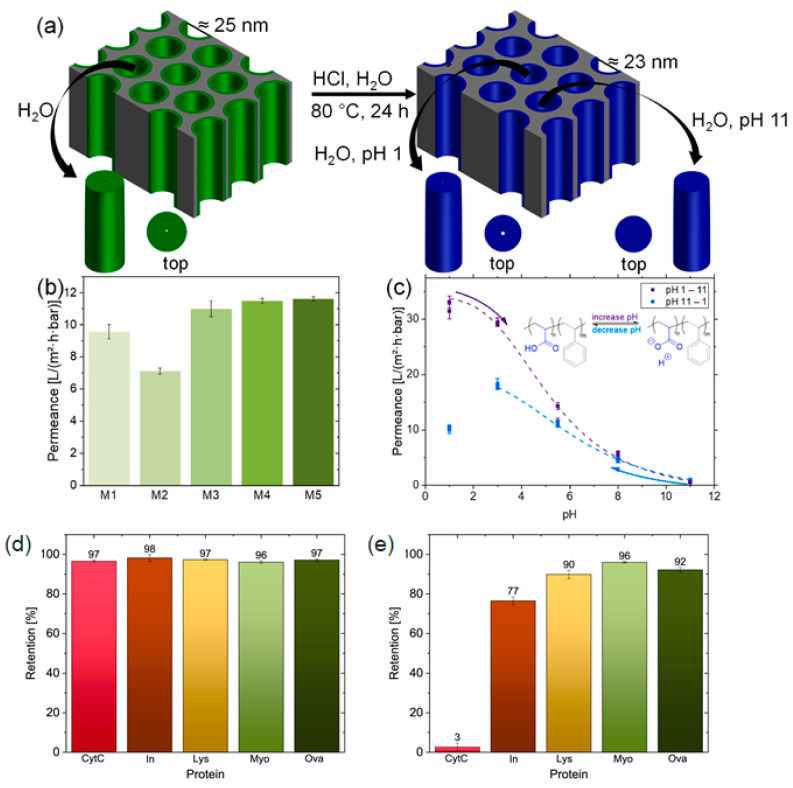
(**a**) Schematic illustration of the PDMA–b–PS membrane in the dried and wet state (**left**) and of the PAA–b–PS membrane (**right**). (**b**) Water permeance of the different PDMA–b–PS membranes (M1–M5). (**c**) pH-dependent water permeance of the PAA–b–PS membrane. (**d**) Protein retention of the pristine PDMA–b–PS membrane and (**e**) PAA–b–PS membrane measured in the dead-end mode in a PBS-buffered system at pH = 7.4. Reprinted with permission from [152], Copyright [2024] by the author (CC BY 4.0).

In addition, Hub et al. recently reported the development of poly(acrylic acid)–block–polystyrene (PAA-b-PS) nanochannels based on poly(N,N-dimethylacrylamide)–block–polystyrene (PDMA-b-PS) with a size of approximately 25 nm in the dry state and approximately 4 nm in the wet state [152] (Figure 5a). Using this nanochannel, they demonstrated selective protein separation according to size and charge state. The proteins used were cytochrome c, insulin, lysozyme, myoglobin, and ovalbumin with different sizes and basic charge states, and cytochrome c could be effectively separated with high selectivity compared to other proteins, as shown in Figure 5e. A target sample for biosensing contains numerous proteins and other substances with different sizes, shapes, and charge states, and complex separation processes, such as electrophoresis and chromatography, and skilled professionals have been required to selectively separate them until now. However, when using nanochannels based on BCPs with high selectivity, the method mainly uses movement restriction according to the size of the target substance, the difference in diffusion, and the difference in affinity through membrane surface modification, so the process is relatively simple compared to existing methods and requires less specialized personnel, so it is expected to be widely utilized in the future.

### 3.3. Surface Structure Control for Biomaterial Signal Amplification

In order to measure the signal of biomaterials such as biomarkers sensitively and reliably, the precise control of the solid electrode surface for signal conversion and measurement is required. In the case of general biomaterial signal measurement such as the electrochemical method, a technique to increase the area of the solid electrode surface can be used to enhance activity, but when using optical measurement techniques such as the Raman and SPR methods, the precise control of the surface structure or the special functional material used becomes more important than the surface area [79].

For example, nanoparticles made of precious metals such as gold have localized surface plasmon resonance (LSPR) characteristics, which means that when the size of the nanoparticle is smaller than the wavelength of the incident light, the energy of the light is absorbed and vibrates [153]. Since this vibrational motion shows different forms depending on the properties of the surrounding materials, it is attracting attention as a very powerful tool for label-free biosensing in the field of biochemistry [154]. Therefore, the uniform dispersion of these precious metal nanoparticles on a solid electrode is a prerequisite for sensitive and reliable signal detection [93].

**Figure 6:**
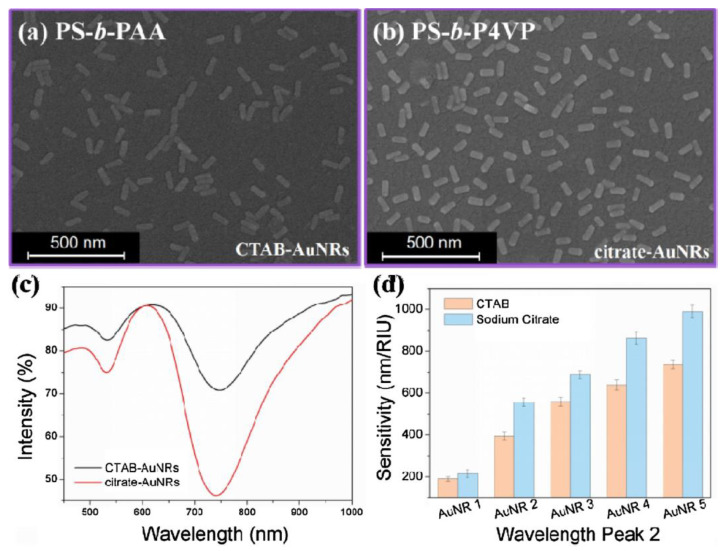
SEM images that show the distribution of AuNR 2 during 12 h of deposition and with (**a**) PS-b-PAA and (**b**) PS-b-P4VP. (**c**) Comparison between the absorption spectra of CTAB-AuNR-based and citrate-AuNR-based LSPR sensors. (**d**) Refractive index sensitivity of the LSPR peak wavelength for different particle sizes. Reprinted with permission from [79], Copyright [2020], Elsevier B.V., Amsterdam, The Netherlands.

The conventional method mainly uses chemical linkers of the silane series, such as 3-aminopropyltriethoxysilane (APTES) and 3-mercaptopropyltrimethoxysilane (MPTMS), but has problems in reliable dispersion, such as the aggregation of nanoparticles. Lu et al. utilized BCPs as cross-linking agents to immobilize noble metal nanoparticles on a solid surface and were able to obtain high dispersion and sensitivity through a simple process [79]. As shown in Figure 6, when poly(styrene)-b-poly(4-vinyl pyridine) (PS-b-P4VP) was used as an intermediate layer for nanoparticle dispersion, the surface morphology was more uniform and showed a higher surface coverage than when conventional alkoxysilanes or polyelectrolytes were used, and the detection limit for human IgG was 0.6 nm, showing very high sensitivity.

In the case of surface-enhanced Raman scattering (SERS) technology, which dramatically improves the detection sensitivity of biomolecules adsorbed on the surface by enhancing the Raman scattering phenomenon on the electrode surface, the roughness control of the electrode surface is important, and for this purpose, metal nanoparticles are mainly adsorbed and used [94,95]. However, unlike the immobilization of nanoparticles on a very flat solid electrode surface such as SiO_2_, this process is complex and difficult in the case of a curved electrode with a sharp needle shape. In this case, BCPs offer great advantages in the immobilization of nanoparticles with high density and dispersion [155].

Zu et al. reported the fabrication of the SERS electrode by immobilizing gold nanoparticles (AuNPs) on glass nanofibers using polystyrene-block-poly(4-vinylpyridine) (PS-b-P4VP) as an intermediate cross-linking material. Compared to the conventional method using 4-mercaptobenzoic acid and a chemical linker, it was possible to fabricate an electrode in which AuNPs were dispersed at a higher density, and a 100% improvement in sensitivity was achieved [135]. In addition, a technology for patterning AuNP monolayers for biosensing platforms with high density and uniformity but no aggregation was recently reported using a simple dip-coating method using PS-b-P4VP, which was the same as in the above study [156].

### 3.4. Precise Control of Biomolecules

As described above, BCPs provide an opportunity to easily achieve fine nanostructures with high precision through self-assembly, unlike complex conventional nanopatterning technologies such as electron beam lithography. The functionality of BCPs is widely used for controllable nanopatterning, but they can also sufficiently perform the role of controlling and monitoring biomolecules such as proteins or DNA. The position, direction, and even the quantity of functional groups contained in individual blocks of BCPs can all be precisely and directly controlled, and this unique structural controllability is similar to that of biomolecules. Biomolecules are precisely manufactured through self-assembly within a biological system, making artificial synthesis very difficult, and the function of the molecule is determined by the position, quantity, and state of the functional groups contained. Therefore, the precise structural control function of BCPs is a great advantage in checking or controlling the function of biomolecules.

Although the technology related to the nanopatterning of BCPs as nanolithography masking materials has been in use for a long time, it is relatively recent that it has been utilized for the control of biomolecules such as proteins. In 2005, Kumar et al. reported a study on the nano-scale control of protein molecules using PS-b-PMMA, a diblock copolymer [96]. They fabricated a nano-scale thin film by periodically alternating two chemical compositions of the diblock copolymer on the surface, and they were able to directionally immobilize proteins at the nano-scale. Since then, various studies have attempted to fabricate proteomic arrays and biosensors using biomolecules such as proteins [97,98,99,157].

**Figure 7:**
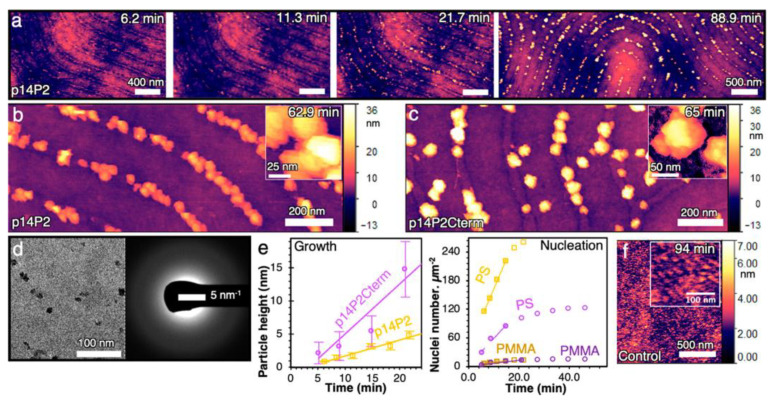
pAmel NR-PS stripes nucleate ACP under a constant chemical potential. In situ AFM shows BCPs that are coated with (**a**) p14P2 nucleates and grow calcium phosphate particles over time. t = 0 min is defined as the time when solution is introduced into the flow cell. (**b**) p14P2 has a high fidelity, and (**c**) p14P2Cterm has a lower fidelity and larger particles. (**d**) Under TEM and SAED, the particles are amorphous. (**e**) A comparison of representative ACP nucleation and growth rates for p14P2 (yellow) and p14P2Cterm (violet). For nucleation rates, the number of nuclei on PS and PMMA are separated and normalized to their corresponding area (Methods in Supporting Information), and the error is the standard deviation. (**f**) The absence of mineralization on BCPs (the control has 12 nm-wide PS stripes and a 24 nm periodicity) without pAmel NR coating even after 94 min. Reprinted with permission from [158], Copyright [2023] by the author (CC BY 4.0).

Recently, Akkineni et al. reported the actual mineralization process of calcium phosphate using PS-b-PMMA thin films as patterning templates to immobilize Amel peptides related to tooth enamel formation [158]. These results demonstrated that the peptides were selectively assembled into the correct conformation in the polystyrene (PS) domain of BCPs and functioned. Figure 7 shows the synthesis process over time. Nuclei gradually grew over time on the nanoribbons (stripe patterns) composed of Amel peptides responsible for calcium phosphate mineralization (a, b, c), which can be confirmed again through the TEM image (d).

**Figure 8:**
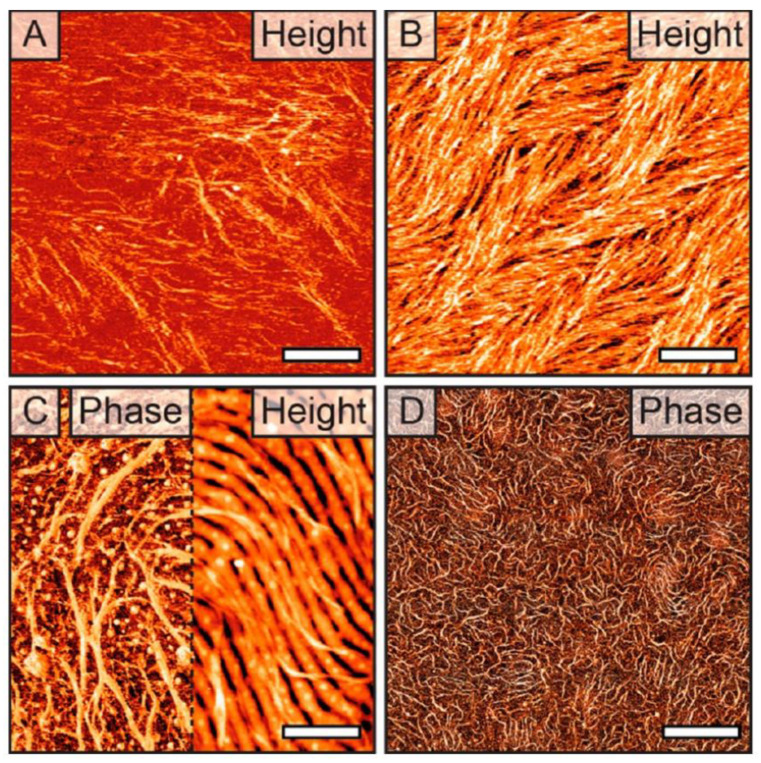
In situ AFM images of collagen at (**A**) 1 and (**B**) 3 μg/mL on mica show self-organization into unidirectional interconnected bundles. (**C**,**D**) In situ AFM images of collagen at 1 μg/mL on PSb-PEO BCP thin films show that bundles of collagen molecules align themselves along the local direction of the underlying BCP substrate. Scale bars in panels (**A**–**C**) represent 200 nm. The scale bar in panel (**D**) represents 2 μm. Reprinted with permission from [159], Copyright [2019], American Chemical Society.

Meanwhile, the results of a study that directly controlled the binding of biomolecules using specific BCPs that were well regulated to have directionality as templates were also reported, which is similar to the mechanism of controlling the assembly or location of proteins at the nano-scale in biological systems [159]. A directional substrate patterned with stripes or dots at the nano-scale was fabricated using polystyrene-block-poly(ethylene oxide) (PS-b-PEO), and using this, the 2D-level directionality control of proteins was performed first, and the binding of collagen, a long and flexible molecule, was controlled secondarily. It was confirmed that the self-assembly speed of the biomolecules was significantly increased on a substrate with alternating chemically distinct nano-scale domains compared to a chemically uniform substrate. As shown in Figure 8, it was confirmed that the growth of the collagen molecules was controlled according to the directionality and properties of the attached substrate. These results imply that BCPs can be applied not only to the control of the 2D crystal growth of proteins using existing BCPs but also to the binding of long, flexible, and complex biomolecules.

### 3.5. Thin Films for Cell Adhesion and Control

It is known that a protein layer fixed on a solid surface has a great influence on various cell operation processes such as the proper attachment, differentiation, and death of cells. Therefore, controlling the arrangement, density, and type of proteins attached to cells can affect not only the control of the cell’s own skeleton, such as cell differentiation, but also the control of cell functions such as gene expression. For example, the molecules commonly used for cell adhesion are mainly collagen, gelatin, or RGD peptide, and the energy required for cell attachment or the environment required for cell growth are controlled through the distribution shape or conditions of these adhesion materials [2,100,101,102].

**Figure 9:**
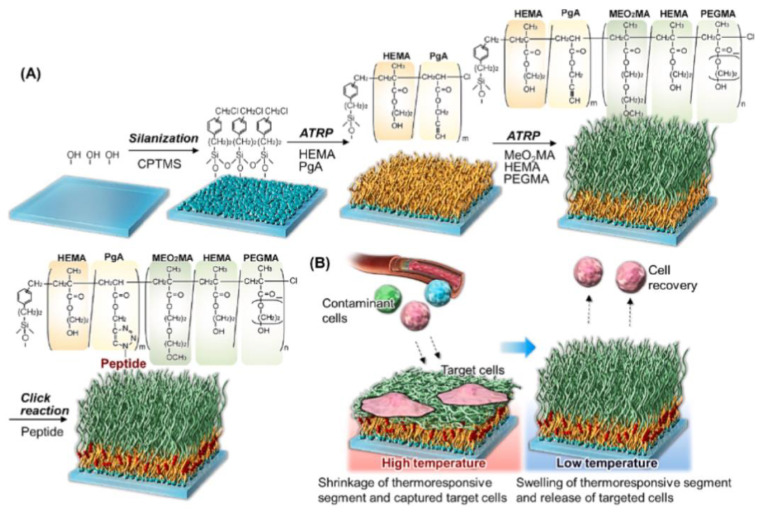
Schematic of selective cell capture by PMEO2MA-based copolymer brushes. (**A**) Preparation of block copolymer brushes with cell-affinity peptides. (**B**) Temperature-modulated separation of vascular cells. Reprinted with permission from [103], Copyright [2023] by the author (CC BY 4.0).

For example, Nagase et al. reported a cell separation process through selective cell adhesion control using ethylene glocol-based BCPS as the main substrate [103]. The fabricated copolymer layer exhibited thermoresponsive properties, and the properties changed according to this thermoresponsiveness to either promote or repel cell adhesion [104,160,161]. By utilizing these properties, adhesion to human umbilical vein endothelial cells (HUVECs) and repulsion to normal human dermal fibroblasts (NHDFs) were observed at 37 °C, and HUVECs attached to the surface could be easily recovered by changing the temperature to 20 °C (Figure 9).

In addition, cell adhesion is greatly affected by the distribution of protein adhesion molecules on the substrate surface, and the nano-scale patterning of the protein molecules is important for controlling structural signaling activity with cells [105]. For example, integrin RGD ligand cell adhesion proteins patterned at the nano-scale have a great effect on cell polarization, migration, and signaling [106], and it has been reported that cell adhesion density is significantly increased on a substrate with a ring-shaped pattern made of PS-b-PI [162]. In addition, a cell culture technology that has antibacterial and sterilizing effects using polystyrene-block-poly(2-vinylpyridine) (PS-b-P2VP) nanopatterns to prevent bacterial infection in cell culture has also been reported [163]. A study was also published on the promotion of the regeneration of attached cells using surface nanopatterns made of BCPs [164]. In that study, the mechanism affecting cell regeneration by promoting cell attachment and subsequent spreading was studied using two types of cells, human bone marrow mesenchymal stem cells (BMMSC) and osteosarcoma cell lines (SaOS-2). As a result of cell culture, the BMMSC promoted attachment and spreading, while the opposite effect was observed in the case of the SaOS-2.

## 4. Other Applications

In addition to the biosensing applications described above, BCPs are widely used in various fields. The most basic application is the field that applies the characteristics of the polymer material itself. Block copolymers, which have the characteristic of repeating a certain structure, can control various physical properties by changing the functional groups of the units that constitute the unit block [107,108]. They can be widely applied to various fields by controlling the strength, glass transition temperature, and tensile strength of the polymer structure [14,109].

In the field of drug delivery, BCPs are being studied in a variety of ways. Since BCPs can structurally form a variety of shapes at the nano-level, they have a structure that is advantageous for delivering a desired substance to a specific location through a certain stimulus. This characteristic can be applied as a sensing platform for nanoimaging or monitoring beyond drug delivery, as they can change their structure by sensing a specific environment or condition [110]. Among the various BCP-based nanostructures for drug delivery, the most commonly used structures are vesicles and micelles. Since the target medium for drug delivery is usually water-soluble, hydrophobic drugs are trapped inside the spheres and moved to the target location. At this time, the hydrophilic region such as PEO exposed to the outside of the vesicle resists the attachment of proteins or cells inside the biological system, preventing enzymatic decomposition or hydrolysis, thereby protecting the drug inside. When the drug delivery vesicle reaches the target location, the vesicle is decomposed using various reaction mechanisms to release the drug inside. Among them, in the case of thermoresponsive BCPs, the internal drug is released through structural transformation when the temperature is above an upper critical solution temperature (UCST) [165,166].

**Figure 10:**
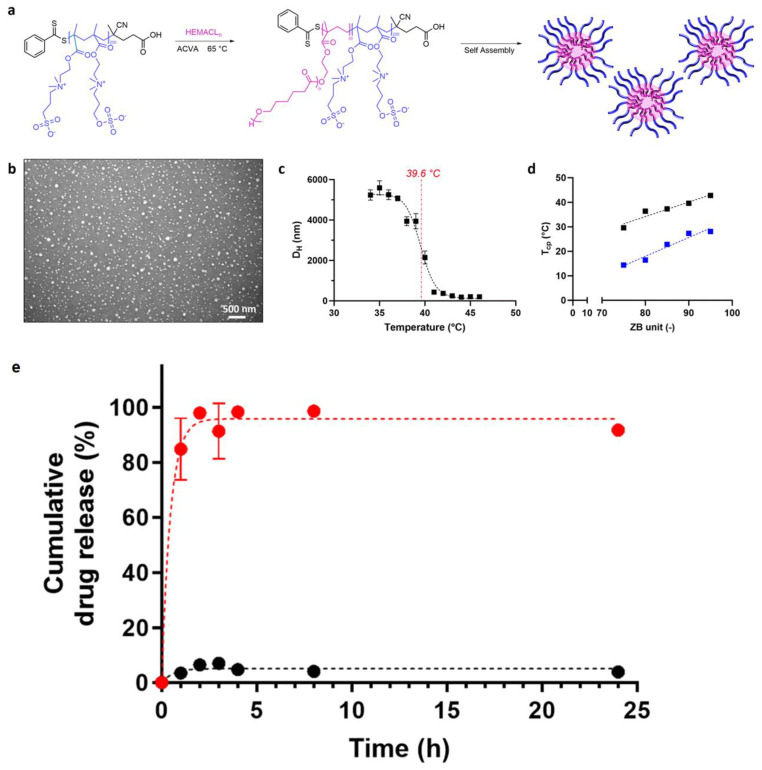
(**a**) RAFT emulsion polymerization using the generic zwitterionic copolymer (DP = 200) as a macromolecular chain transfer agent and HEMACLn as a monomer and the consequent NP formation. (**b**) TEM image of the 110-90-5 NPs recorded by staining the warm polymer suspension above its Tcp. (**c**) Representative curve of the hydrodynamic diameter of the NPs as a function of the temperature. (**d**) Correlation between the Tcp of the NPs and the units of ZB in the zwitterionic portion of the polymer. (**e**) Release profiles of PTX delivered by p(110SB-co-90ZB)-b-HEMACL5 NPs at 43 °C (red) and 37 °C (black), measured by HPLC. Reprinted with permission from [167], Copyright [2024] by the Royal Society of Chemistry (CC BY-NC 3.0).

For example, Colli et al. recently reported the fabrication of thermoresponsive drug delivery nanoparticles based on poly(sulfobetaine-co-sulfabetaine) (p(SB-co-ZB)) with extended chains [167] (Figure 10a). The drug delivery of paclitaxel, a common anticancer drug, was tested using the nanoparticles. While more than 95% of the drug was retained under the normal physiological conditions of 37 °C, most of the drug was released under conditions of 43 °C, which satisfies the requirement of hyperthermia, demonstrating that the nanoparticles are an effective drug delivery system (Figure 10c,e).

In addition to thermoresponsive BCPs, drug delivery technologies utilizing various reaction mechanisms such as pH, redox, light, and enzymes have been studied [111,112,113,168,169]. For example, Zhang et al. recently reported the development of multifunctional BCPs nanoparticles that can deliver drugs in response to heat, pH, and light [170]. The drug contained in the nanoparticles showed a release rate of more than 80% within 3 h under UV irradiation and a 50 °C environment, and it was confirmed that the release rate was accelerated in an alkaline environment. In addition, although photoresponsive BCPs usually depend on short-wavelength excitation such as UV, which limits drug release in tissues, the development of BCPs that react with long-wavelength visible light has also been recently reported [114].

Another application of BCPs in the biological field is biocatalysis. The promotion of chemical reactions through part or all of the operating system of a biological system is called biocatalysis, and enzymes are representative biocatalysts. Natural enzymes are widely used in various industries due to their high activity and substrate specificity, but they have many difficulties due to their high cost and low stability. Therefore, many studies have been conducted to mimic artificial enzymes with cheaper and more stable structures. Since the report of ferromagnetic nanoparticles with activity similar to natural peroxidase in 2007, many studies have been conducted in the field of nanozymes, which refer to nanoparticles with enzymatic properties [115,116]. At this time, if BCPs with an artificially controllable nano-level arrangement are utilized for the composition and operation of the above-mentioned biocatalysts, a wide variety of research attempts become possible. For example, Qu et al. constructed an artificial peroxidase using hemin, a type of porphyrin containing chloride and iron, and poly(ethylene glycol)-block-poly(4-vinylpyridine) (PEG-b-P4VP) [171]. The hemin was located inside the micelle structure of the amphiphilic core–shell structure of BCPs, showing a biocatalytic effect, which is similar to the working principle of actual peroxidase, and showed excellent catalytic performance. In addition, many similar studies using BCPs as building blocks have been conducted [172], and recently, Huang et al. reported a study on a targeted treatment platform for hypoxic cancer using a BCPs-based nanozyme [173]. By utilizing the structural change in BCPs with a pH response at the target site, they decomposed hydrogen peroxide and generated oxygen through an artificial enzymatic reaction, showing excellent targeting ability.

In addition, BCPs such as poloxamer with biocompatibility can be used to fabricate biomaterials for various applications that come into contact with biological systems, such as drug delivery carriers or platforms for cell attachment, as described above [174]. Additional applications include tissue engineering related to the treatment and reconstruction of damaged cells and tissues [175]. The platforms used here should have mechanical properties above a certain strength and biocompatibility [176] and microscopic environmental controllability for cell growth and differentiation for regeneration [177], and BCPs can provide such environments [178,179,180]. In addition, 3D printing application studies utilizing the molding properties of the polymer itself are also noteworthy [181], and the fabrication of biomimetic scaffolds using BCPs as inks for 3D bioprinting has been reported [182]. In addition, gene therapy and delivery [183,184] and in vivo imaging studies [185] are also receiving much attention.

This review describes recent biosensing studies based on diblock and triblock copolymers. These studies have utilized the differences in the components of immiscible blocks to induce uniform repeating structures, which are then applied to biosensing platforms. Meanwhile, multiblock copolymers (MCPs) with an increased number of blocks have recently attracted much attention, as they are novel polymers that exhibit very different properties and behaviors from conventional diblock and triblock copolymers [186]. MCPs can be used primarily to directly generate hybrid structures using various polymer wastes that are immiscible, which can play a significant role in the recycling of plastic wastes [187] and can also be utilized in the development of new materials through the introduction of new structures [188]. In addition, in the biofield, MCPs can provide great opportunities for the fabrication of various nanostructures that mimic biological systems due to their complex structural features [189,190]. For example, MCPs can provide more differentiated properties than conventional di- and triblock copolymers and thus can be used in various fields such as biosensors, drug delivery, and catalysis [191,192,193]. However, although much research has been conducted on the self-assembly process that creates the complex structures of MCPs [194,195], there are still many aspects that are not understood, and thus much research is needed in the future [196].

## 5. Conclusions and Perspectives

Many researchers have made efforts in the past to elucidate many principles related to the synthesis and structure control of BCPs based on self-assembly. BCPs technology has been applied to not only traditional fields such as thermoplastic elastomers but also various applications requiring the control of molecules and materials at the nano-level. In particular, the technology has been widely used in the biosensor field and produced greater synergy effects when combined with existing top–down technology. BCPs, which can control shape and arrangement at the molecular level and expand the arranged molecular structure to the bulk level through continuous bonding, can provide great advantages as a biosensing platform. For the development of biosensors, biomaterials perform functions at the molecular level. To perform such functions, the control of the reactivity of specific parts in the three-dimensional structure is necessary. For this reason, BCPs can function as a powerful biosensing platform since they can control the type of functional group or the level of intermolecular bonding in smaller molecular sequences than macromolecules such as proteins and enzymes.

Although BCPs have been developed as a biosensing platform and have various advantages, many tasks still need to be studied. First, we need to go beyond the current nanostructure manufacturing technology to create more sophisticated nanostructures. In addition, we need to gain a deeper understanding of the strength and flexibility of the structure by adjusting the interaction between the unit blocks that make up the polymer. Moreover, we need to expand the precise control to the 3D level beyond the simple repetition of two or three types of identical units. As a biosensing platform, the interaction between a single type of biomolecule and the BCPs interface has been studied significantly, but the interaction with multicomponent biomolecules is still being explored, and more effort is needed when targeting cells. In summary, future research should be directed to develop more complex BCPs-based nanostructured biomaterials that require a better understanding of interface interaction with two or more biomolecules. In addition, since many of the studies have not been applied to actual industries, we need to focus on strengthening practical usability by strengthening research collaboration with companies from the research stage.

## Figures and Tables

**Table 1:** Types of biosensing platform applications and the roles of BCPs.

Application Types	Key Features	The Role of BCPs	References
Electrochemical Biosensors	Redox reaction, electrical conductance changes	Immobilization platform (reaction area, activity, efficiency)	[62,63,64,65,66,67,68]
Optical Biosensors	Changes in optical properties	Immobilization platform Nano/micro array platform Environmental responsiveness	[69,70,71,72,73,74,75,76,77,78,79]
Piezoelectric Biosensors	Resonant frequency changes	Affinity control	[80,81,82]
Calorimetric Biosensors	Temperature changes	Immobilization platform (reaction area, activity, efficiency)	[83,84,85]
Nanopatterning	Lithography technology	Self-assembly-based masking layer	[44,46,47,48,49,50,51,54,55,56,57,58]
Nanochannels	Nano-scale porous channels	Material transfer platform	[86,87,88,89,90,91,92]
Nanoarray	Nano-level surface arrangement control	Uniform surface array platform	[77,79,93,94]
Protein Array and Chip	Controlling the arrangement of biomolecules	Immobilization platform	[95,96,97,98,99]
Cell Array and Chip	Regulation of cell adhesion and differentiation	Immobilization platform	[100,101,102,103,104,105,106]
Drug Delivery	Optional structural changes	Selective responsive platform	[107,108,109,110,111,112,113]
Catalyst	Biocatalysis	Artificial enzyme structure	[114,115,116]

## Data Availability

Not applicable.
